# Integration between ROS Regulatory Systems and Other Signals in the Regulation of Various Types of Heat Responses in Plants

**DOI:** 10.3390/ijms19113370

**Published:** 2018-10-28

**Authors:** Kazuma Katano, Kohey Honda, Nobuhiro Suzuki

**Affiliations:** Department of Materials and Life Sciences, Faculty of Science and Technology, Sophia University, 7-1 Kioi-cho, Chiyoda, Tokyo 102-8554, Japan; ktnkzm0519@gmail.com (K.K.); kohey-jyochi@eagle.sophia.ac.jp (K.H.)

**Keywords:** acquired thermotolerance, basal thermotolerance, Ca^2+^, heat shock transcription factor, heat stress memory, nitric oxide, reactive oxygen species, stress combination, systemic acquired acclimation

## Abstract

Because of their sessile lifestyle, plants cannot escape from heat stress and are forced to alter their cellular state to prevent damage. Plants, therefore, evolved complex mechanisms to adapt to irregular increases in temperature in the natural environment. In addition to the ability to adapt to an abrupt increase in temperature, plants possess strategies to reprogram their cellular state during pre-exposure to sublethal heat stress so that they are able to survive under subsequent severe heat stress. Such an acclimatory response to heat, i.e., acquired thermotolerance, might depend on the maintenance of heat memory and propagation of long-distance signaling. In addition, plants are able to tailor their specific cellular state to adapt to heat stress combined with other abiotic stresses. Many studies revealed significant roles of reactive oxygen species (ROS) regulatory systems in the regulation of these various heat responses in plants. However, the mode of coordination between ROS regulatory systems and other pathways is still largely unknown. In this review, we address how ROS regulatory systems are integrated with other signaling networks to control various types of heat responses in plants. In addition, differences and similarities in heat response signals between different growth stages are also addressed.

## 1. Introduction

Even short periods of heat stress can dramatically affect almost all aspects of plant growth and development, and reproduction. Heat stress can, therefore, have detrimental effects on yield production worldwide and cause devastating economical and sociological impacts. Indeed, it was demonstrated that heat stress caused 5.5% and 3.8% reductions in global yields of wheat and maize, respectively, over the past decades (1980–2008) [1]. In addition, climate prediction models suggested that the increase in the global surface temperature might exceed 2 °C by the end of 21st century (Intergovernmental Panel on Climate Change 2014). It is, therefore, urgent to develop plants and crops with enhanced heat tolerance to fulfill future food demand for the increasing population worldwide.

Because of their sessile lifestyle, plants cannot escape from heat stress and are forced to alter their cellular state to prevent damage. Plants, therefore, evolved various strategies to adapt to irregular increases in temperature. For instance, plants possess the ability to survive under abrupt temperature increases. This ability of plants is known as basal thermotolerance [2,3,4]. In addition, plants are able to cope with lethal heat stress following pre-exposure to sublethal heat stimuli and subsequent recovery under optimal temperature [2,3,4]. For this heat acclimatory response, referred to as acquired thermotolerance, the recovery phase following pre-exposure to sublethal heat stimuli might be essential for re-programming of the cellular state [5,6]. This duration, called the “heat memory” phase, can range from hours to days, or even generations [7,8]. Furthermore, when a small part of a plant is locally exposed to heat stress, heat response mechanisms are activated in distal systemic tissues that are not directly exposed to heat stress [9,10]. The activation of heat response mechanisms in systemic or non-challenged tissues, termed systemic acquired acclimation (SAA), might be essential to prevent further damage to the entire plant [11]. Moreover, plants need to adapt to the simultaneous occurrence of multiple biotic or abiotic stresses in the natural environment. Given the recent climate prediction model showing a future increase in global surface temperature, it is expected that biotic and abiotic stresses, as well as heat stress, will have increasingly detrimental effects on crop production in the future [12,13]. Recent studies demonstrated that responses of plants to combined stresses are different from that to each corresponding single stress [14,15].

These heat stress responses in plants are regulated by networks of various pathways. For instance, a network of heat shock transcription factors (HSFs) is highly conserved and is one of the most studied pathways underlying heat responses in plants. HSFs function as transcriptional regulators that bind to a defined heat shock element (HSE) in the promoters of heat response genes, including heat shock proteins (HSPs), essential molecular chaperones that maintain proper protein folding under stress conditions [16,17]. An HSF-dependent pathway was considered to be involved in both basal and acquired thermotolerance [2,4,17]. Multiprotein bridging factor 1c (MBF1c) is another key regulator of heat response that might function independently of the pathways involving several major HSPs, such as HSP70, 90, and 101 [3]. MBF1c plays an important role in basal thermotolerance via controlling the trehalose, salicylic acid, and ethylene signaling pathways during heat stress [3]. In addition, plant hormone signals, including abscisic acid (ABA), salicylic acid (SA), jasmonic acid (JA), and ethylene, also play important roles in regulating heat responses in plants [18,19,20]. A previous study employing various mutants deficient in key regulators of different signaling pathways revealed the significance of different hormones in the regulation of basal and acquired thermotolerance in plants [2]. Furthermore, ABA was shown to be involved in long-distance signaling activated by heat stress [10], as well as the response of plants to heat stress combined with drought or salinity [21,22].

The significance of reactive oxygen species (ROS) and nitric oxide (NO) regulatory systems in heat stress responses in plants was also evidenced in numerous studies. Although ROS have long been considered as toxic byproducts that cause damage to cells, recent studies demonstrated the important roles that ROS play as signaling molecules to regulate a broad range of biological processes underlying growth, development, and biotic/abiotic stress responses [23]. Plants, therefore, possess a large network of ROS-producing/scavenging pathways involving more than 100 genes to strictly maintain the appropriate cellular ROS level [23,24]. Disruption in ROS scavenging systems resulted in enhanced sensitivity of plants to heat stress, suggesting the important roles of these systems for the protection of plants against heat stress [2,25,26,27]. However, ROS-producing systems were also shown to be required for the activation of HSF-dependent pathways and long-distance signals during heat stress [10,28,29]. These findings indicate that a delicate balance between ROS production and scavenging is essential for heat responses in plants. Nitric oxide (NO) recently gained increasing attention from plant researchers due to its involvement in the response of plants to various abiotic stresses [30,31]. NO also functions as a key signaling molecule that mediates various responses of plants to heat stress [31]. For instance, the antioxidant function of NO under abiotic stresses, including heat stress, was well documented in previous reports [30,31,32,33]. NO might control cellular redox homeostasis through its ability to neutralize the harmful ROS. For instance, NO reacts with O_2_^−^ to produce NO_3_^−^, resulting in the prevention of excess H_2_O_2_ accumulation [4]. In addition, NO was also shown to modulate the activity of enzymatic and non-enzymatic antioxidant systems in plants under heat stress [30,31]. In addition to integration with ROS scavenging systems, NO might also function together with H_2_O_2_-dependent signals. A previous study demonstrated that NO might act downstream to H_2_O_2_ signaling to regulate HSF-dependent pathways under heat stress [34].

Based on these findings, we can hypothesize that multiple pathways might be strictly coordinated to regulate various types of heat responses in plants. However, the complex mode of coordination between multiple pathways to tailor the cellular state for the various heat responses still needs to be elucidated. In this review, we dissect signal networks underlying different types of heat responses in plants based on recent findings, especially through the perspective of integration of ROS regulatory systems with other signals. We also address different mechanisms of heat response functions in different tissues and developmental stages. Most of the molecular mechanisms introduced in this review were found from through analyses of *Arabidopsis*. However, the information might be extended to other plant species.

## 2. Cross-Talk between ROS and Other Signals Involved in the Regulation of Basal Thermotolerance in Seedlings and Vegetative Stage

ROS play a key role as signaling molecules that regulate growth, development, defense, and abiotic stress acclimation, despite their toxic potential. Plants, therefore, possess large and precise ROS regulatory systems involving both ROS-producing and -scavenging pathways to maintain appropriate cellular ROS levels [23,24]. Several lines of evidence revealed the significance of ROS regulatory systems in the heat stress response of plants during the vegetative stage [2,35]. More than a decade ago, Larkindale and co-workers tested the basal and acquired thermotolerance in seedlings of *Arabidopsis* mutants deficient in different pathways, including ROS regulatory systems and hormone signaling [2]. This assessment of heat tolerance demonstrated that ROS metabolism mutants deficient in antioxidant pathways were more defective in basal thermotolerance than acquired thermotolerance, especially in the presence of high light. In addition, deficiency in ascorbate peroxidase 1 (APX1), a cytosolic ROS-scavenging enzyme, resulted in enhanced sensitivity to heat stress and growth retardation [36,37]. Furthermore, a more recent study demonstrated that transgenic tobacco plants over-expressing wheat F-box protein exhibited enhanced basal thermotolerance accompanied by the higher activity of ROS-scavenging enzymes and a lower level of ROS accumulation [38]. These results indicate that ROS-scavenging systems play essential roles for regulating the basal thermotolerance of plants by preventing the accumulation of toxic levels of ROS.

In *Arabidopsis*, five putative heat sensors localized in different cellular compartment were identified. These sensors include a Ca^2+^ channel, transcription factors regulating unfolded protein responses in the endoplasmic reticulum (ER-UPR) and cytosol (CPR), a histone sensor in the nucleus, and red/far-red light sensors in the chloroplast and nucleus [4,39,40,41,42,43]. Among these putative heat sensors, a deficiency in cyclic nucleotide gated channel 2 (CNGC2), a putative unit of the Ca^2+^ channel, in *Arabidopsis* resulted in enhanced basal thermotolerance accompanied by a slight increase in H_2_O_2_ accumulation [44]. This slight increase in H_2_O_2_ might function as a signal to trigger APX-dependent heat response pathways that prevent the accumulation of toxic levels of excess H_2_O_2_. Such a modulation of H_2_O_2_ level might lead to the enhanced tolerance of roots and leaves to lethal heat stress in CNGC2-deficient mutants [44]. In addition, CNGC2-deficient mutants also showed a higher accumulation of cytosolic Ca^2+^ under heat stress [41]. The integration between Ca^2+^ signaling and ROS regulatory systems was strongly evidenced in previous studies [45,46]. For instance, Ca^2+^ is required for the activation of O_2_^−^-producing reduced nicotinamide adenine dinucleotide phosphate (NADPH) oxidase, respiratory burst oxidase homologs (RBOHs) which possess Ca^2+^-binding EF-hand motifs [46,47,48]. These findings indicate that ROS acting as signaling molecules produced via Ca^2+^ signaling might also play important roles in the protection of plants against heat stress. It should, therefore, be necessary to analyze the activity of ROS-producing systems in CNGC2-deficient mutants in future studies. Nevertheless, in contrast to CNGC2-deficient mutants, a deficiency in myeloblastosis 30 (MYB30) transcription factor, which might negatively regulate annexins and Ca^2+^ signals, resulted in impaired basal thermotolerance, despite the higher level of cytosolic Ca^2+^ [49]. Thus, the effects of enhanced cytosolic Ca^2+^ level on heat tolerance could be dependent on the state of other pathways.

Hormone signals are also known to be integrated with ROS regulatory systems during heat stress [50,51]. A recent study demonstrated that ethylene response factor 74 (ERF74) in *Arabidopsis* was shown to be a positive regulator of respiratory burst oxidase homolog D (RBOHD), a ROS-producing enzyme [52]. Overexpression of ERF74 resulted in enhanced basal thermotolerance, whereas mutants deficient in ERF74 displayed the opposite phenotype. ERF74 might regulate the expression of the *RbohD* gene by binding to its promoter. Thus, it should also be interesting to address how ethylene and Ca^2+^ signaling are integrated to activate RBOHD. ABA is another plant hormone that might function together with ROS regulatory systems. An RNA-binding protein, flowering control locus A (FCA), regulates basal thermotolerance via interaction with ABA-insensitive 5 (ABI5), a transcription factor known to regulate expression of antioxidant genes, including 1-cysteine peroxiredoxin 1 (PER1) [53]. In addition, APX6 was shown to protect germinating seeds against abiotic stresses including heat stress via mediating cross-talk between ROS regulatory systems, ABA signaling, and auxin [54]. MBF1c, a key player in basal thermotolerance, functions upstream to salicylic acid and ethylene signaling, as well as trehalose signaling [3]. An MBF1c-dependent pathway was previously proposed to function independently of pathways involving ROS regulatory systems and several major HSPs. However, our recent study demonstrated that *Arabidopsis* deficient in CNGC2 showed a higher accumulation of MBF1c protein under heat stress during the seedling stage, suggesting that CNGC2-dependent Ca^2+^ signaling and ROS regulatory systems might regulate MBF1c pathways [44]. The integration of MBF1c and Ca^2+^ signaling could also be supported by a recent study showing that the overexpression of Antarctic moss MBF1c in *Arabidopsis* plants resulted in enhanced salt tolerance, accompanied by the activation of ROS-scavenging systems, maintenance of Adenosine triphosphate (ATP) homeostasis, and facilitation of Ca^2+^ signaling [55]. These findings indicate the existence of a complex network involving MBF1c, hormone signals, Ca^2+^ signals, and ROS regulatory systems.

Tight links between NO and ROS regulatory systems under abiotic stress were also evidenced in many studies. NO functions as an important signaling molecule that regulates the post-translational modification of proteins. Once NO production is induced in response to abiotic stress, *S*-nitrosylation, the attachment of an NO group to a protein thiol (SH) of the cysteine (Cys) residue to form an *S*-nitrosothiol (SNO), occurs [30,56,57]. Several proteins involved in responses of plants to abiotic stresses are targeted by NO-induced *S*-nitrosylation [30]. Under heat stress, NO signaling might play key roles in regulating various processes such as the maintenance of photosynthetic apparatus, activation of antioxidant enzymes, membrane protection, and stomatal movement. For example, the exogenous treatment of NO donors on tall fescue plants resulted in an improvement in photosystem II (PSII) performance, accompanied by attenuated oxidative damage and ROS accumulation under heat stress [32]. In maize seedlings, hydrogen sulfide (H_2_S) was shown to be a signaling molecule functioning downstream of NO in the regulation of basal thermotolerance [58]. NO signaling mediated by H_2_S might play a key role in membrane protection and the attenuation of oxidative damage during heat stress [33]. Furthermore, NO was also shown to mitigate heat-induced oxidative damage via the neutralization of harmful ROS and the modulation of carotenoids [31,59].

Although NO was shown to activate ROS-scavenging systems, positive integration between NO and ROS signaling was also reported. Interestingly, NO was shown to function downstream of H_2_O_2_ signaling in the regulation of basal thermotolerance [34]. Seedlings of *Arabidopsis* mutants deficient in respiratory burst oxidase homologs B and D (RBOHB and RBOHD) were impaired in heat-induced NO accumulation. The heat-sensitive phenotype of RBOH-deficient mutants was rescued upon the application of NO donors or the overexpression of genes involved in NO synthesis. In addition, NO might act upstream to Ca^2+^ signals during heat stress [34,35], although Ca^2+^ signaling was shown to be required for the activation of RBOHs [46,48]. These results indicate the existence of a feedback loop to modulate ROS, NO, and Ca^2+^ signaling during heat stress. Furthermore, the signaling cascade involving ROS, Ca^2+^, and NO might regulate heat stress responses by stimulating the binding of HSFs to target DNA [31,60].

**Figure 1:**
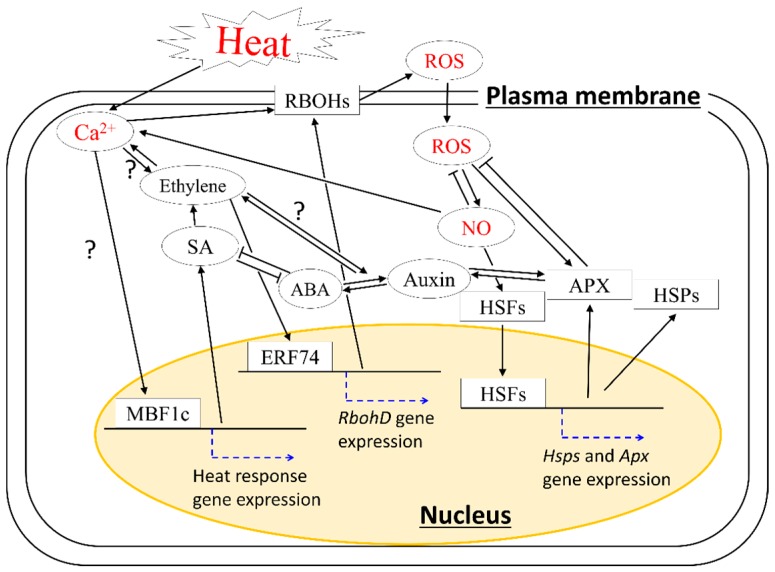
Simplified model of signaling pathway governing basal thermotolerance in plants. Important pathways involving heat shock proteins (HSPs), reactive oxygen species (ROS)-scavenging enzymes (such as ascorbate peroxidases (APXs)), hormones, and multiprotein bridging factor 1c (MBF1c) might be modulated by a feedback loop of Ca^2+^, ROS, and nitric oxide (NO). Rectangles indicate proteins; ellipses indicate other signaling molecules, e.g., Ca^2+^ ions, hormones, ROS, and NO; black arrows indicate positive regulation; T-shaped lines indicate negative regulation; blue dotted arrows indicate activation of gene expression; yellow circle indicates nucleus. Heat stimuli and key signaling molecules (Ca^2+,^ ROS and NO) are indicated in red characters. Names of cellular components are indicated in underlined characters. The pathways that can be hypothesized but not clearly evidenced in previous studies are marked with “?”.

Taken together, these findings suggest that important pathways involving HSPs, ROS-scavenging enzymes (such as APXs), plant hormones, and MBF1c-dependent signals might be strictly modulated by Ca^2+^, ROS, and NO signals. The integration of heat response pathways underlying basal thermotolerance is summarized in Figure 1.

## 3. Acquired Thermotolerance in Seedling and Vegetative Stage

**Figure 2:**
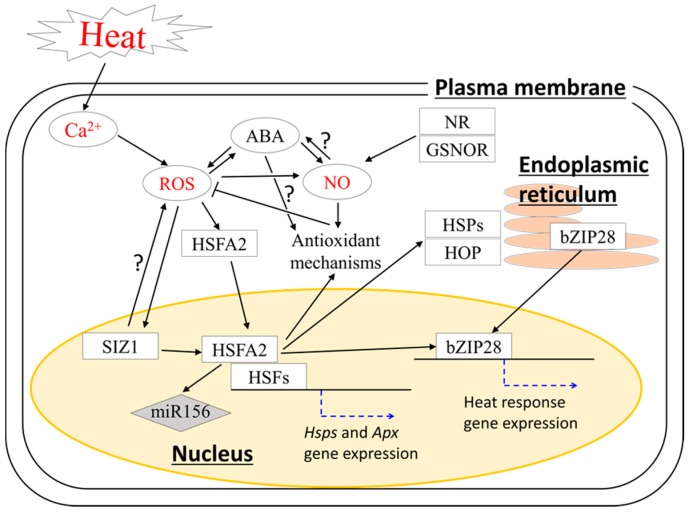
Simplified model of signaling pathway governing acquired thermotolerance in plants. Heat shock transcription factors (HSFs), including heat shock transcription factor A2 (HSFA2), modulated by a network of ROS, Ca^2+^, and NO signaling, might function as master regulators of this pathway. HSFA2 might be also regulated by small ubiquitin-like modifier (SUMO)ylation via the function of SUMO E3 ligase (SIZ1). Basic leucine zipper protein 28 (bZIP28) might act as a downstream signal of HSFA2 activated by ROS signaling. Rectangles indicate proteins; the gray diamond indicates microRNA (miRNA); ellipses indicate other signaling molecules, e.g., Ca^2+^ ions, ABA, ROS, and NO; black arrows indicate positive regulation; T-shaped lines indicate negative regulation; blue dotted arrows indicate activation of gene expression; yellow circle indicates the nucleus; orange ovals indicate the endoplasmic reticulum. Heat stimuli and key signaling molecules (Ca^2+^, ROS and NO) are indicated in red characters. Names of cellular components are indicated in underlined characters. The pathways that can be hypothesized but not clearly evidenced in previous studies are marked with “?”.

The exposure of plants to sublethal heat stimuli can result in an improved ability of plants to activate faster and stronger responses to subsequent severe heat stress. Such an ability to adapt to severe heat stress, i.e., acquired thermotolerance, evolved in plants to survive in the natural environment. The pathways underlying acquired thermotolerance were also uncovered in previous studies (Figure 2). The differences in protein requirements and transcriptome expression between basal and acquired thermotolerance suggested that different pathways might be involved in triggering these different heat responses in plants [4,27]. The differences in the pathways underlying these heat responses could also be supported by the heat response phenotypes of mutants deficient in different pathways [2]. The assessment of basal and acquired thermotolerance using seedlings of *Arabidopsis* mutants demonstrated that the mutant deficient in ABA signaling exhibited the strongest defects in acquired thermotolerance [2]. ABA was shown to be highly increased during the initial phase of pre-exposure of plants to sublethal heat stimuli to enhance acquired thermotolerance [19]. Contrarily, mutations in ROS-producing enzymes, ABA biosynthesis, and salicylic acid (SA) accumulation resulted in weaker defects in acquired thermotolerance [2]. In addition, cytosolic APX2, heat shock transcription factor 7a (HSFA7a), and nuclear factor-X1 (NF-X1) were identified as key regulators of acquired thermotolerance in *Arabidopsis* [27]. Although *Arabidopsis* mutants deficient in APX2 were impaired in both basal and acquired thermotolerance during the seedling stage [3,27], the impairment of acquired thermotolerance was more severe compared with that of basal thermotolerance. *Arabidopsis* plants deficient in APX2 demonstrated a lower survival rate by approximately 15% compared to wild-type (WT) plants when seedlings were directly exposed to lethal heat stress (i.e., basal thermotolerance) [26]. However, the survival rate of this mutant was lower than that of WT plants by 70–80% when seedlings were pre-treated with sublethal heat stimuli before being subjected to lethal heat stress (i.e., acquired thermotolerance) [27]. These results indicate that APX2 might be more important for acquired thermotolerance. In addition, a deficiency in HSFA7a or NF-X1 also resulted in an impairment of acquired thermotolerance at a level similar to that of mutants deficient in APX2 [27].

Among putative heat sensors, ER-UPR and CPR were shown to regulate brassinosteroid signaling and HSPs, respectively, under heat stress [40,42]. HSFA2, a key regulator of CPR, is required for acquired thermotolerance, as well as for the activation of ROS-scavenging systems [6,29]. HSFA2 is known as a key mediator of H_2_O_2_ signaling and heat stress response in plants [28,29]. In addition, several substances, such as transcription factors, HSPs, and microRNAs (miRNAs), might also play essential roles in acquired thermotolerance regulated by HSFA2. HSP70 and HSP90 were shown to be required for the regulation of the DNA-binding activity and stability of HSFs during pre-exposure of plants to sublethal heat stimuli to enhance acquired thermotolerance [61]. A recent study revealed that the HSP70–HSP90 organizing protein (HOP) family might play a key role in acquired thermotolerance through direct interaction with HSP90 in *Arabidopsis* [62]. Furthermore, miR156, which is targeted by HSFA2, was also shown to contribute to acquired thermotolerance by downregulating the expression of a transcript encoding SQUAMOSA-promoter binding like (SPL) transcription factor via post-transcriptional modification [63]. A recent study demonstrated the integration of basic leucine zipper protein 28 (bZIP28), a key regulator of the ER-UPR pathway, with HSFA2 and ROS signaling [64]. Deficiency in bZIP28 in *Arabidopsis* resulted in enhanced expression of the *HsfA2* transcript, accompanied by higher accumulation of H_2_O_2_, as well as of HSPs and APX proteins. These results indicate that the dysfunction of the bZIP28-dependent pathway might be compensated by the activation of H_2_O_2_ signaling and HSFA2-dependent pathways. In contrast, deficiency in HSFA2 resulted in the inhibition of *bZip28* transcript levels, as well as in a lower accumulation of H_2_O_2_, indicating that HSFA2 might function upstream to bZIP28 under heat stress. In this heat response pathway, H_2_O_2_ signaling might act upstream to HSFA2, because the role of HSFs in the sensing of H_2_O_2_ was proposed in previous studies [28,29]. Furthermore, the role of small ubiquitin-like modifier (SUMO) E3 ligase (SIZ1) in the regulation of HSFA2 was also demonstrated in tomato [65]. HSFA2 might be upregulated by SUMOylation via the function of SIZ1. These results indicate that HSFA2 might be one of the master regulators of acquired thermotolerance that govern multiple pathways.

Although differences in the pathways underlying basal and acquired thermotolerance were indicated in previous findings, we still cannot ignore the possibility that these different heat responses might also share common mechanisms. Indeed, CNGC2-deficient mutants in *Arabidopsis* showed enhanced acquired thermotolerance [41], as well as basal thermotolerance [44]. The involvement of cross-talk between Ca^2+^ and ROS signaling in both basal and acquired thermotolerance could be supported by the enhanced level of cytosolic Ca^2+^ and H_2_O_2_ accumulation in CNGC2-deficient mutants during heat stress [41,44]. NO might be another player regulating both basal and acquired thermotolerance. The level of *S*-nitrosoglutathion (GSNO), a reservoir of NO, is known to be regulated by GSNO reductase 1 (GSNOR1) under heat stress [66]. A recent study demonstrated that *Arabidopsis* plants deficient in GSNOR1 exhibited impaired acquired thermotolerance, accompanied by enhanced NO_3_^−^ and SNO levels, as well as defects in reproductive growth [67]. These results indicate that the maintenance of a proper NO/SNO ratio might be essential in the regulation of acquired thermotolerance.

In plants, NO production under environmental stress might also be induced by nitric oxide synthase (NOS)-like activity that depends on nitrate reductase (NR), as well as l-arginine and polyamines [31,68,69]. NR-dependent NO was shown to activate antioxidant enzymes via H_2_O_2_ and ABA signaling [70]. A recent study demonstrated that NO synthesis via the function of NR is dependent on the direct interaction between NR and nitric oxide-forming nitrite reductase (NOFNiR), which belongs to the amidoxime reducing component (ARC) protein family in *Chlamydomonas reinharadtii* [71,72]. NOFNiR can convert nitrite to NO using electrons from NADPH via the diaphorase activity of NR. *Arabidopsis* has two genes coding for the ARC protein, and one of them presents NO-producing activity in vitro [72,73]. Although NR-dependent NO production was shown to be enhanced in response to various abiotic stresses, the response of NR activity to heat stress was not obvious in some species such as pea and *Pleurotus eryngii* var. *tuoliensis* [68,69]. Thus, the significance of NR-dependent NO in the regulation of heat stress responses is still not clear. However, NO produced by this pathway might function together with ABA [31], a key regulator of acquired thermotolerance [2]. Therefore, we cannot ignore the possibility that NR-dependent NO production could be involved in acquired thermotolerance.

These results indicate that, to some extent, basal and acquired thermotolerance are regulated by common players. However, the mode of coordination between these players might be differently modulated.

## 4. Stress Memory and Systemic Acquired Acclimation; Recently Emerged Heat Acclimatory Responses in Plants

**Figure 3:**
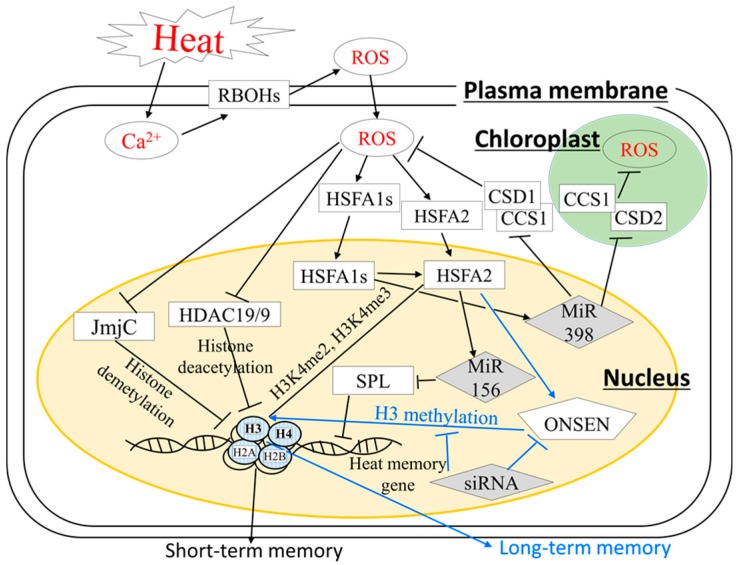
Simplified model of signaling network underlying heat stress memory. Histone methylation, histone acetylation, and miRNA-dependent signals might be key processes for maintaining high expression levels of heat stress memory genes. These key processes could be modulated by ROS, NO, and Ca^2+^. Rectangles indicate proteins; gray diamonds indicate microRNAs; the pentagon indicates the ONSEN transposon; blue ellipses indicate histones; white ellipses indicate other signaling molecules, e.g., Ca^2+^ ions, hormones, ROS, and NO. Pathways regulating short- or long-term memory are indicated with black or blue arrows, respectively. Black arrows indicate positive regulation; T-shaped lines indicate negative regulation; yellow circle indicates the nucleus; green circle indicates the chloroplast. Heat stimuli and key signaling molecules (Ca^2+^, ROS and NO) are indicated in red characters. Names of cellular components are indicated in underlined characters.

For efficient acquired thermotolerance, stress memory, an ability of plants to remember past exposure to stress to be better prepared for subsequent severe stress, is essential [7]. This area of research only recently received increasing attention [74,75,76,77]. Although the expression of many genes can be enhanced in response to heat stress, the expression of only certain sets of genes (memory genes) maintains a very high level for several hours or even days following recovery from heat stress. This maintenance of memory gene expression was accompanied by chromatin remodeling, histone modification, and heat-inducible transposons [78] (Figure 3). A recent study demonstrated that HSFA2, shown to be activated by heat-induced ROS production, might recruit methyltransferase to heat response genes to sustain their expression [8]. In addition, sustained expression of these memory genes was associated with sustained accumulation of histone H3 lysine 4 trimethylation and dimethylation (H3K4me3 and H3K4me2), which persisted even after active transcription from the loci subsided. In plants and animals, H3K4 trimethylation, associated with the active transcription of genes, might be required for the proper function of RNA polymerase [79,80,81,82]. Furthermore, epigenetic changes might be associated with heat-induced programmed cell death (PCD) in leaves of maize [83]. Heat stress can induce the accumulation of O_2_^−^, leading to PCD, associated with an increased level of histone acetylation. These results suggest that heat stress memory might be modulated via the cross-talk between ROS signaling and histone modification. Moreover, plants possess mechanisms to regulate heat stress memory via the activation of a transposon known as ONSEN [84]. ONSEN might be positively regulated by small interfering RNA (siRNA)-mediated pathways; however, siRNA biogenesis-deficient mutants showed a high frequency of new ONSEN insertion into heat stress memory genes following heat stress. These heat-induced ONSEN insertions in this mutant were shown to be conserved in the second generation of stressed plants [84]. HSFA1s and HSFA2 might act as activators of ONSEN under heat stress. These HSFs possess a highly conserved N-terminal DNA-binding domain (DBD) that is required for binding to HSE in the long terminal repeat existing in the ONSEN promoter [85]. Taken together, these results suggest that HSFA2, a key regulator of ROS signaling, might play an essential role in the activation of multiple signals involved in heat stress memory. This hypothesis might be reasonable because HSFA2 was also suggested as a key regulator of acquired thermotolerance (Figure 2). It should be necessary to address how ROS regulatory systems are integrated with histone methylation- or ONSEN-dependent heat stress memory.

Post-transcriptional regulation by miRNA may also play a key role in the activation of heat stress memory. For instance, a previous study demonstrated that miR156 might activate heat stress memory through the downregulation of SQUAMOSA-promoter binding like (SPL) transcription factors, negative regulators of anthocyanin biosynthesis [63,86]. Furthermore, miR398, which contains a putative HSE in its promoter region, was shown to be directly targeted by HSFA1s, and it regulates heat stress memory by repressing the expression of genes encoding copper/zinc superoxide dismutase 1 and 2 (CSD1 and 2) and copper chaperone for superoxide dismutase 1 (SOD1) (CCS), which dismutate O_2_^−^ to H_2_O_2_ in the cytosol and chloroplasts [87]. In this signaling cascade regulated by HSFA1s, the inhibition of CSD1, CSD2, and CCS might lead to further ROS accumulation, and a positive loop of ROS generation can be accelerated [87].

ROS signals that function independently of the HSFA2 pathway might also regulate heat stress memory via histone acetylation [88]. Histone acetylation activates the expression of genes that govern growth and development under stress conditions [89]. For instance, histone acetyltransferase 2 (HAF2) is required for the integration between light signal and acetylated histone to activate light-response gene expression. In addition, HAF2 was shown to be involved in chlorophyll accumulation under optimal light conditions to regulate proper growth [90]. ROS repress the expression of histone deacetylation 19/9 (HDAC19/9), leading to the acetylation of all lysine residues in the N-terminal tails of histone proteins and the induction of downstream gene expression [88]. Furthermore, ROS might directly or indirectly repress Jumonji C (JMJC), a flavin adenine dinucleotide (FAD)-dependent demethylation enzyme, to induce stress response genes [88,91]. A recent study demonstrated that FORGETTER1 (FGT1) was also shown to loosen chromatin structure to sustain high expression levels of heat stress memory genes [92]. FGT1 might bind to the promoter of genes encoding small HSP-related genes [92]. Further studies are required to uncover the relationship between FGT1 and HSFs.

A systemic response, depending on the propagation of long-distance signaling, might be another essential process required for the proper regulation of acquired thermotolerance. The systemic response of plants to pathogen infection (systemic acquired resistance (SAR)), or wounding was extensively studied in a network of numerous signals [93,94]. In contrast, a new type of systemic response, termed systemic acquired acclimation (SAA), recently emerged as an important acclimation response of plants to abiotic stresses [10,95]. The concept of SAA is based on the concept of SAR, which is well known. Therefore, many players involved in SAA and SAR overlap extensively. However, in this review, we mainly focus on the mechanisms underlying SAA. For more details on SAR, we would like to refer our readers to more extensive reviews (see References [11,94]).

When a small group of cells in a plant are exposed to abiotic stimuli, long-distance signals can be propagated to distal parts of the plant that are not directly exposed to stimuli [9,11,96]. A recent study demonstrated that SAA activated by heat stress was effective for the protection of entire plants against lethal heat stress [10]. The auto-propagation of long-distance signals was shown to be regulated by a RBOHD-dependent ROS wave that might be directly linked to a Ca^2+^ wave and probably to an electric signal [96]. The ROS wave, a “universal signal”, might integrate with other pathways to establish stress-specific signals [10,11]. For instance, an ROS wave activated by the local application of high light was accompanied by the accumulation of photorespiratory amino acids, including glycine and serine, in tissues not directly exposed to high light [10]. On the other hand, a local heat stimulus was able to activate an ROS wave that integrated with ABA signals [10]. The direct link between heat-induced ROS waves and ABA was also evidenced upon finding that mutants deficient in ABA synthesis or signaling were deficient in heat-induced SAA, in addition to the propagation of ROS waves [10].

Recent findings on SAR focused on the interdependence of ROS signaling on NO. The *noa1*/*nia1* double mutant, deficient in NO accumulation, demonstrated almost completely compromised SAR, as well as lower ROS accumulation, in systemic tissues that were rescued by exogenous application of H_2_O_2_ [96,97]. In addition, RBOHD was shown to be regulated via an NO-dependent process in response to pathogen attack [98]. Although these findings indicate that NO functions upstream to RBOH-dependent ROS signals in defense responses, *Arabidopsis* lacking RBOHD or respiratory burst oxidase homolog F (RBOHF) were impaired in the accumulation of NO in response to pathogen attack [97,99]. These results suggest that the balance between ROS and NO might be modulated via a feedback loop regulating SAR. The involvement of such cross-talk between ROS waves and NO signals in heat-induced SAA is yet to be elucidated. It should, therefore, be interesting to study the coordination between NO signaling and ROS waves in heat-induced SAA. Indeed, the requirement for NO signaling in the heat response of plants was evidenced in previous findings [30,31]. In addition, NO-mediated *S*-nitrosylation was also shown to inhibit H_2_O_2_-scavenging enzymes such as APX and catalase (CAT) [35,100], suggesting that NO could also promote H_2_O_2_ accumulation by reducing its decay, leading to the acceleration of ROS waves.

Taken together, these findings indicate the significance of ROS signals in the regulation of heat memory and SAA activated by heat stress. However, the mode of coordination between ROS signals and other pathways in these newly emerged heat acclimatory responses is still poorly understood. Mechanisms underlying these heat responses should, therefore, be analyzed in mutants deficient in various ROS regulatory systems in future studies.

## 5. Heat Response in Reproductive Stage

**Figure 4:**
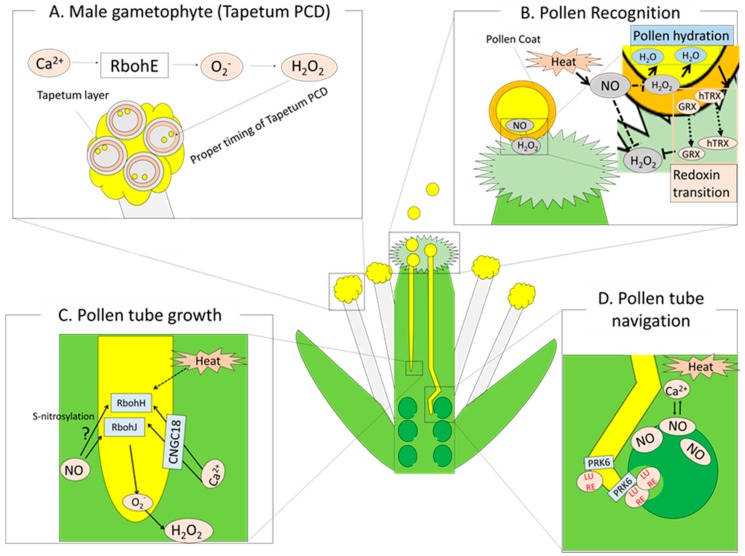
Integration between ROS, NO, and Ca^2+^ signals in reproductive tissues that can be affected by heat stress. The models indicate the signals that function in different reproductive developmental processes. (**A**) Tapetum programmed cell death (PCD) occurs in anthers. Respiratory burst oxidase homolog E (RbohE) regulated by Ca^2+^ is essential to generate ROS that accelerate PCD. (**B**) Pollen–pistil interaction occurs on stigma. Redoxin proteins such as H-type thioredoxins (hTRXs) and glutaredoxins (GRXs), which accumulate in pollen grains and pollen coats, are rapidly released to papilla cells on the top of stigma. (**C**) Pollen tube elongation inside style. ROS produced by respiratory burst oxidase homologs H and J (RbohH and J) in the tip of pollen tubes might be key regulators of this process, (**D**) Pollen tube navigation toward ovules. NO in ovules modulated by Ca^2+^ signals might function as a negative chemotraffic substance for pollen tube navigation to prevent inappropriate guidance. LURE and pollen receptor-like protein kinase 6 (PRK6) function to properly guide pollen tube elongation. Green and yellow colors indicate female and male reproductive tissues, respectively; small yellow particles in (**A**) indicate pollens; the dotted arrows in (**B**) indicates transfer of molecules; the green circle in (**D**) indicates ovule. Black arrows indicate positive regulation; T-shaped lines indicate negative regulation. The pathways that can be hypothesized but not clearly evidenced in previous studies are marked with “?”.

Deciphering the response of reproductive tissues to heat stress is considered as a major research avenue because heat-induced damage on reproductive tissues can directly impact on yield of important crops such as maize, rice, wheat, and soybeans. Vegetative and reproductive tissues might share common heat response signals such as HSP networks, ER-UPR, CPR, Ca^2+^ signaling, and ROS regulatory systems [101]. Because of the specific mechanisms of reproductive development, however, it is expected that plants might also develop specific mechanisms to protect male gametophytes (pollen and anther development), female gametophytes (stigma development), and processes of pollination and fertilization. Such specificity of the heat response pathway in reproductive tissues could also be supported by the findings that different sets of HSPs (small HSPs, HSP70, HSP90, and HSP101) accumulate in developing anther and pollen grains in response to heat stress, whereby small HSPs in particular accumulate in reproductive tissues [101,102,103]. Here, we mainly focus on the cross-talk between ROS, NO, and Ca^2+^ signaling, which plays an important role in the protection of these developmental processes in reproductive tissues against heat stress (Figure 4).

### 5.1. Heat Stress Response during Pollen Development

Prior to the opening of flowers, the maturation of pollen grains proceeds in anthers via microsporogenesis and microgametogenesis. A pollen mother cell undergoes meiosis to generate four microspores. Mature pollens are then generated from microspores through mitosis. These processes were shown to be sensitive to heat stress. For example, meiosis II spindle orientation was altered by heat stress, leading to the generation of abnormal diploid gametes instead of normal haploid in the meiotically restituted dyad or tetrad [104]. During the maturation of pollen grains, nutrients provided by the tapetum are necessary to construct the pollen wall. For the development of viable pollens, proper timing of tapetal degeneration is also required. Tapetum programmed cell death (PCD) occurs when pollen mitotic division occurs. This proper timing of tapetum degeneration through PCD was shown to be progressed by RBOH-dependent ROS production in *Arabidopsis* and rice [105,106] (Figure 4A). A deficiency or overexpression of respiratory burst oxidase homolog E (RBOHE) in *Arabidopsis* caused improper timing of tapetal degeneration due to an alteration in the timing of tapetum PCD, resulting in pollen abortion [105]. Despite the requirement of ROS-dependent PCD in proper pollen development, excess PCD in pollen might also be a main cause of disturbance of plant reproduction during heat stress [104]. Pollen and tapetal cells were shown to contain a large number of mitochondria, one of the main sources of ROS production [107]. Thus, the imbalance between mitochondrial ROS production and scavenging pathways could result in abnormal PCD in tapetum during heat stress [107].

A recent study demonstrated that deficiency in CNGC2 in *Arabidopsis* resulted in enhanced Ca^2+^ influx into the cytosol under heat stress [41]. Despite enhanced heat tolerance in seedlings, *Arabidopsis* deficient in CNGC2 showed a reduction in seed production under heat stress when compared with WT plants [44]. Higher heat sensitivity in the seed production of this mutant might be due to the oxidative stress caused by the enhanced ROS production. These results suggest that the modulation of cytosolic Ca^2+^ is essential for the maintenance of proper levels of ROS to protect reproductive tissues against heat stress. CNGC2 is also known as defense no death 1 (DND1), which is required for the activation of PCD under pathogen attack [108]. It should, therefore, be interesting to address whether deficiency in CNGC2 also affects the RBOH-dependent tapetum PCD required for pollen development during heat stress [18].

These findings indicate that a proper regulation of ROS level is essential for the maintenance of proper pollen development under heat stress. Interestingly, ROS signals trigger ER-UPR pathways, as well as HSPs and ROS-scavenging systems, to protect pollen development against heat stress [101]. The significance of the ER-UPR pathway in the maintenance of pollen development was evidenced in a recent study. The deficiency in increased organ regeneration 1 (IRE1) in *Arabidopsis*, resulting in a loss of function of alternative spliced bZIP60-dependent UPR regulation, led to pollen abortion under heat stress caused by the alteration of pollen coat properties [109].

### 5.2. ROS and Redox Regulation during Pollination and Fertilization

After the production of mature pollen grains followed by anther dehiscence, mature pollen grains land on the stigma and complete pollination through three steps of pollen–pistils interaction: pollen adhesion, hydration and germination, and pollen tube growth.

Several studies indicated that NO–ROS redox signaling plays a crucial role in pollen–pistil interaction [45,110] (Figure 4B). Prior to and during the arrival of pollens to pistil, high levels of NO and H_2_O_2_ accumulation were observed in the pollen and stigma, respectively [110,111]. The high accumulation of NO and ROS might activate signals triggering H_2_O_2_ scavengers in the stigma following pollen hydration [45]. In a previous study, *Arabidopsis* plants deficient in homolog of yeast sucrose nonfermenting 4 (SNF4), a component of the sucrose nonfermenting 1-related protein kinase 1 complex in the vegetative cells of pollen grains, showed impaired pollen hydration and germination on the stigma and a decrease in ROS accumulation in pollens [112]. Pollens of SNF4-deficient mutants also showed impaired biogenesis and ultrastructure of the mitochondria and peroxisomes. These results suggest that SNF4 might be required for the regulation of ROS signaling via functions of the mitochondria and peroxisomes in pollen vegetative cells to induce pollen hydration and germination [112]. Following pollen hydration, redoxin proteins such as H-type thioredoxins (hTRXs) and glutaredoxins (GRXs), which accumulate in pollen grains and pollen coats, are rapidly released; then, papilla cells on the top of stigma are filled with these redoxins [110]. These redoxins might attenuate ROS levels in papilla cells on the stigma. Although the effects of heat stress on these processes are still poorly understood, we should not discard the possibility that heat stress might disturb these pollen–pistil interaction processes because of the heat sensitivity of ROS and NO regulatory systems [31]. It should be necessary to address how NO signals regulate the pathways involving these redoxins to support pollen tube penetration into the stigma during heat stress.

Once pollen grains germinate, pollen tube elongation initiates by utilizing nutrients supplied from the stigma. The elongation and navigation of pollen tubes also require ROS production induced by Ca^2+^ in the tip of tubes [113,114,115] (Figure 4C). In *Arabidopsis*, respiratory burst oxidase homolog H and J (RBOHH and RBOHJ) activated by Ca^2+^ in germinated pollens and the tip of pollen tubes are essential for proper pollen tube elongation [115]. In addition, a recent study demonstrated that deficiency in CNGC18, a Ca^2+^ channel on the plasma membrane, in *Arabidopsis* plants resulted in disturbed pollen germination and pollen tube growth [116]. Furthermore, mitochondrial Ca^2+^ regulated by the mitochindaria calcium uniporter complex (MCU) expressed in vegetative cells of pollen grains and pollen tubes was also shown to function as an additional player required for pollen tube growth [117]. To achieve successful fertilization, plants also take advantage of NO–ROS redox signaling for the navigation of pollen tubes toward the micropyle of ovule [110,118] (Figure 4D). In contrast to ROS, which positively regulate pollen tube elongation, NO functions as a negative chemotraffic substance for pollen tube navigation [119]. Prado and co-workers demonstrated that NO production was detected in the whole ovule, except for the micropyle. To prevent the incorrect guidance of pollen tube elongation, NO might be recognized by Ca^2+^ in the pollen tubes, and tube elongation might be correctly directed toward the micropyles that lack NO accumulation [119]. Pollen tube elongation and navigation are known to be sensitive to heat stress [120,121]. In addition, heat stress might disturb this pollen tube guidance process due to the heat-induced alteration of NO generation [31]. Several players, such as LURE1, pollen receptor-like protein kinase 6 (PRK6), and rho of plant guanine nucleotide exchange factors (ROPGEFs), are known to be positive attractants of pollen tubes toward micropyles [122,123]. However, the integration of these factors with ROS and NO regulatory systems are still not elucidated.

Ca^2+^, ROS, and NO signals might be common players that regulate multiple processes of reproductive development. However, specific mechanisms within each process could function to protect reproductive tissues against heat stress, because key players that regulate each developmental process might be different. It should, therefore, be necessary to identify these key players and their integration with Ca^2+^, ROS, and NO signals in each developmental process.

## 6. Response of Plant to Heat Stress Combined with Other Abiotic Stresses

The responses of plants to various abiotic stresses that negatively impact growth and development in the field, such as drought, heat stress, cold stress, and salinity, were extensively studied [12,124,125,126]. However, the field environment in nature is very different from the controlled conditions employed in previous researches, because multiple abiotic stresses can simultaneously occur [14,15]. In addition, a recent climate prediction model suggested that the temperature increase might exceed 2 °C by the end of 21st century. We can, therefore, expect that heat stress simultaneously occurring with other biotic or abiotic stresses might cause detrimental effects on crop yield in the future. Furthermore, several transcriptome analyses demonstrated that unique mechanisms governing the responses of plants to stress combinations could not be easily predicted from studies focusing on individual stress factors [127,128,129], supporting the necessity of research focusing on stress combinations.

As in the case of single stress, ROS-detoxifying proteins might be essential for protecting plants against oxidative damage caused by combined stresses. A previous study employing different citrus species reported that Carrizo citrange was more tolerant to a combination of heat stress and drought compared to Cleopatra mandarin [130,131]. The higher tolerance of Carrizo citrange to this stress combination might be associated with the increase in SOD, CAT (Catalase), APX, and glutathione reductase (GR) activity, as well as the maintenance of a favorable glutathione to glutathione disulfide (GSH/GSSG) ratio. In addition, Carrizo citrange showed higher PSII efficiency and lower oxidative damage compared to Cleopatra mandarin [131]. These results suggest that the maintenance of photosynthetic apparatus via the modulation of chloroplastic ROS levels might be essential for protecting plants against this stress combination. Furthermore, a higher accumulation of SA was observed in Cleopatra mandarin under a combination of heat stress and drought. In contrast to SA, which highly accumulated in response to a combination of heat stress and drought, ABA, which was shown to antagonize SA signaling [132], decreased in both citruses under this stress combination.

The integration of ROS regulatory systems with other mechanisms, such as the maintenance of photosynthetic apparatus and hormone signaling, need to be strictly modulated in the response of plants to a combination of heat stress and drought. The behaviors of ROS regulatory systems and hormone signaling were shown to be differently modulated depending on plant species. For example, in lotus, catalase activity only increased upon the combination of heat stress and drought. However, in clover, the activity of this enzyme increased relative to the control upon heat, drought, and combined stress [133]. In addition, a decline in SA accumulation was observed in *Arabidopsis* upon the combination of heat stress and drought [21], although SA accumulation increased in citruses in response to the same stress combination. A recent study demonstrated that *Arabidopsis* plants deficient in isochorismate synthase 1 (ICS1), named *sid2-1*, showed higher tolerance to a combination of heat stress and drought [134]. ICS was shown to be required for SA synthesis, as well as for the production of phylloquinone, one of the components of the PSI complex that mediates the electron transfer from PSI to ferredoxin [135]. Under a combination of heat stress and drought, *sid2-1* plants showed a higher accumulation of PSII reaction-center proteins, accompanied by the enhanced expression of transcripts involved in repairing these proteins. In addition, *sid2-1* plants accumulated higher levels of H_2_O_2_ compared to WT plants under this stress combination. These results suggest that the modulation of signals associated with SA synthesis might be essential for the maintenance of photosynthetic machineries and ROS regulatory systems under a combination of heat stress and drought. It is still not understood how photosynthetic apparatus can be targeted by or protected against oxidative stress under this stress combination in the *sid2-1* mutant. Furthermore, the expression of genes encoding ROS-scavenging proteins targeted to the chloroplast and cytosol was increased under a combination of heat stress and drought, whereas the expression of mitochondrial genes was decreased in this stress combination in date palm [136]. It should, therefore, be necessary to elucidate the mode of coordination between ROS regulatory systems and other pathways that function in different organelles under a combination of heat stress and drought.

*Arabidopsis* plants showed a dramatic increase in the accumulation of ABA under a combination of heat stress and drought, although ABA accumulation declined in citruses under the same stress combination [21,131]. As a signaling molecule, ABA was shown to play a pivotal role in tailoring the response of plants to various stress combinations via integration with other hormones and ROS regulatory systems [137]. In *Arabidopsis*, mutants deficient in ABA synthesis or signaling showed higher sensitivity to a combination of heat stress and drought [21], suggesting the significance of ABA in the response of plants to this stress combination. Interestingly, ABA is required for the expression of APX1 and MBF1c proteins, which are essential for the response of *Arabidopsis* plants to this stress combination [138,139]. ROS are known to affect ABA biosynthesis and signaling, thereby accelerating Ca^2+^ influx into stomatal guard cells and modulating stomatal closure under drought [140,141]. However, under a combination of heat stress and drought, H_2_O_2_, rather than ABA, might be essential for stomatal movement [21].

The response of plants to a combination of heat stress and salinity is another process that requires ABA signaling. A recent study demonstrated that *Arabidopsis* plants deficient in ABA synthesis or signaling were more sensitive to a combination of heat stress and salinity [22]. Regarding antioxidants, the concentration of flavonols that were recently described as powerful antioxidants [142,143] was much higher under heat stress than under the combination of salinity and heat [144].

Taken together, these findings indicate that the coordination between ROS regulatory systems and hormone signals might be differently modulated depending on the type of stress combination and plant species. It should be important to find master regulator(s) governing such different coordinations between ROS and hormone signals under stress combinations.

## 7. Conclusions

In the natural environment, plants are exposed to various patterns of temperature increase. Numerous researches attempted uncovering the molecular and physiological mechanisms that govern the various types of heat stress responses in plants. In this review, we addressed ROS regulatory systems underlying these heat stress responses and their integration with other pathways. Based on the previous findings, we can suggest that these different types of heat responses might be regulated by different mechanisms. The involvement of different mechanisms in the various types of heat responses could also be supported by the findings from comprehensive analyses of transcripts, proteins, and mutants [2,27]. In addition, each different heat response could be regulated by different key regulator(s). For example, MBF1c, which functions upstream to SA, ethylene, and trehalose, was shown to be required for basal thermotolerance [3]. In contrast, HSFA2, which mediates H_2_O_2_ signal and heat response mechanisms [28], might be a key regulator of acquired thermotolerance [6,29]. Interestingly, HSFA2 might also contribute to the maintenance of heat stress memory, supporting the significance of this transcription factor in acquired thermotolerance. Heat stress responses might also be different depending on growth stages and types of tissues. The development of reproductive tissues is regulated by specific ROS–NO cross-talk, which is distinct from the mechanisms governing vegetative growth [45,105,106,110]. The specific mechanisms of reproductive development might be disturbed by heat stress, and counteracting mechanisms to prevent damage to reproductive tissues caused by heat stress might also be regulated by specific mechanisms. Furthermore, when other abiotic stresses simultaneously occur with heat stress, responses of plants to these combined stresses are also different from those to the corresponding single stresses [14,15].

Differences in the mechanisms underlying different types of heat stress responses might also be, at least partially, attributed to differences in the patterns of signaling networks between various pathways. ROS, NO, and Ca^2+^ might function as common regulators involved in the different types of heat stress response. However, the mode(s) of coordination between these common regulators and other pathways might be different depending on the types of heat stress. In addition, the source of ROS generation might be different depending on the types of stress and tissues. Indeed, the stress- or tissue-dependent specificity of RBOH functions was proposed [48]. For instance, respiratory burst oxidase homolog D (RBOHD) was shown to be required for the auto-propagation of long-distance signaling [10]. In contrast, RBOHE, H, and J are involved in different steps of reproductive development [105,115]. It should, therefore, be necessary to elucidate specific regulators that govern these different sources of ROS generation.

## Abbreviations

ABA	Abscisic acid
APX	Ascorbate peroxidase
bZIP	Basic leucine zipper protein
CAT	Catalase
CNGC	Cyclic nucleotide gated channel
CPR	Cytosolic unfolded protein response
ER	Endoplasmic reticulum
ER-UPR	Endoplasmic reticulum unfolded protein response
GR	Glutathione reductase
GSNO	*S*-nitrosoglutathion
GSNOR	GSNO reductase
HSE	Heat shock element
HSF	Heat shock transcription factor
HSP	Heat shock protein
ICS	Isochorismate synthase
JA	Jasmonic acid
MBF1c	Multiprotein bridging factor 1c
NO	Nitric oxide
NR	Nitrate reductase
PCD	Programmed cell death
ROS	Reactive oxygen species
